# Crystallise, poise, capture: a multimodal platform for correlated structural and spectroscopic characterisation of redox enzymes

**DOI:** 10.1007/s00775-026-02148-x

**Published:** 2026-05-05

**Authors:** Shoba Laxmi, Sofia Jaho, William K. Myers, Kylie A. Vincent, Stephen B. Carr

**Affiliations:** 1 Department of Chemistry, Inorganic Chemistry Laboratory, University of Oxford, South Parks Road, Oxford, UK; 2 Rutherford Appleton Laboratory, Research Complex at Harwell, Harwell Campus, Didcot, UK; 3 Diamond Light Source, Harwell Science and Innovation Campus, Didcot, UK; 4 Centre for Advanced Electron Spin Resonance (CAESR), Department of Chemistry, University of Oxford, Oxford, UK

**Keywords:** Ferredoxin, Electrochemistry, Metalloprotein, Crystal, X-Ray diffraction, EPR

## Abstract

Iron–sulfur (Fe-S) clusters are ubiquitous as redox-active protein cofactors, but it is often difficult to collect protein structures in which redox centres are in uniform and well-defined oxidation states. Using spinach ferredoxin I (Fdx) as a model redox protein, we demonstrate an integrated methodological pathway for electrochemical modulation of redox state in protein crystals coupled with in crystallo EPR and online-UV-visible spectroscopy to verify oxidation state. We show that Fdx crystals can be electrochemically reduced, reversibly, without compromising lattice integrity or X-ray diffraction quality. We show that redox levels can be precisely ascertained in crystallo via EPR and UV-visible spectroscopy, enabling a direct correlation between protein structure and electronic state of the metal cluster. In this way, we generate and compare ’oxidised’, ‘reduced’ and ‘re-oxidised’ structures of Fdx. Overall, our approach demonstrates a pipeline which will be applicable to structure-function studies of a wide range of electron-transfer proteins and redox enzymes.

**Graphical abstract:**

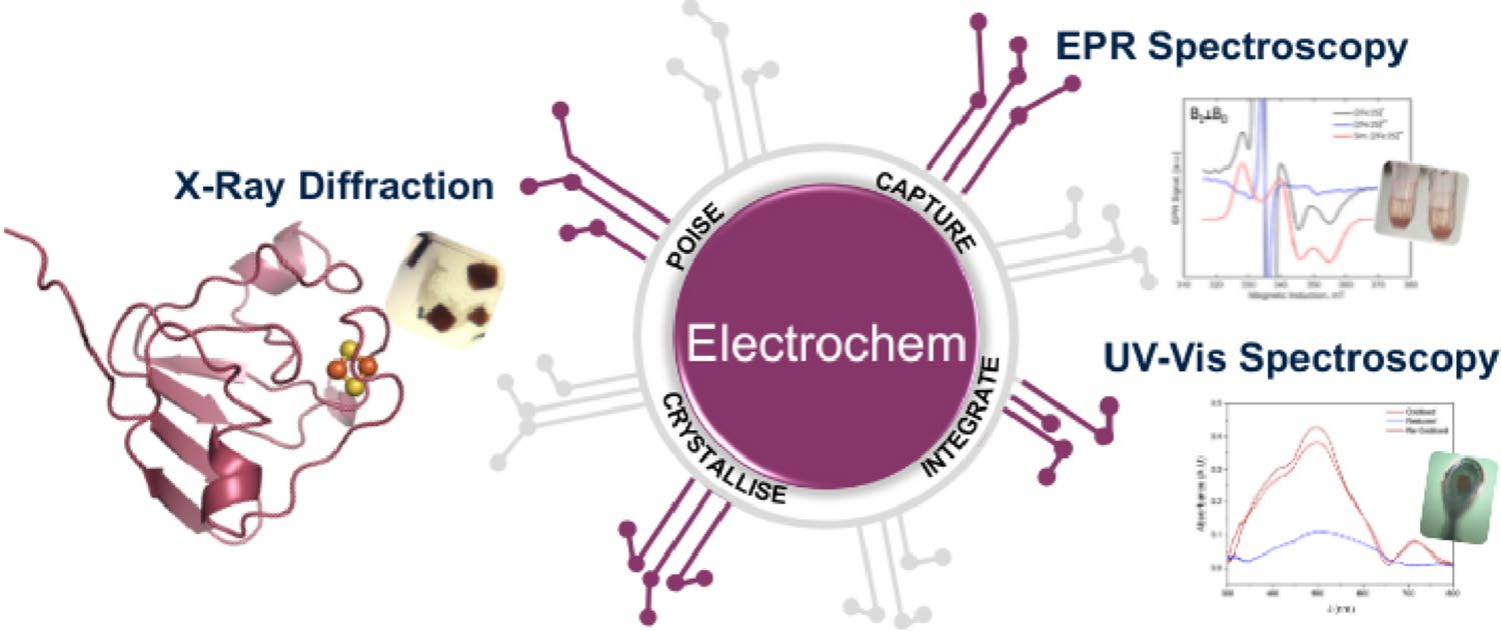

**Supplementary Information:**

The online version contains supplementary material available at 10.1007/s00775-026-02148-x.

## Introduction

Iron–sulfur (Fe–S) clusters are an evolutionarily conserved class of metallocofactors, essential for the function of many proteins across all domains of life. Their biochemical versatility stems from structural diversity in both the cluster composition and its interactions with the surrounding protein. Fe-S clusters are typically composed of [2Fe-2S], [3Fe-4S] or [4Fe-4S], and they are typically covalently attached to the protein via cysteine thiolate ligands, but are also found coordinated by a growing set of alternative residues: histidine [1], aspartate [2] arginine [3], glutamate [4] and alcohols serine [5] and threonine [6], as well as substrates and co-factors [7]. The resulting variation in composition, geometry, ligand coordination and surrounding protein environment finely tune their redox potential [8] and reactivity, resulting in numerous physiological roles, including electron transport, catalysis, gene regulation and redox sensing [9–13]. Ferredoxins are a prominent family of Fe-S proteins that mediate critical one-electron transfer processes that drive diverse metabolic reactions ranging from nitrogen fixation [14] to lipid desaturation [15].

The efficiency of ferredoxins as electron transport agents is predicated on the structural rigidity of their metal centres, where the protein scaffold imposes strict geometric constraints [16–19] to minimise reorganisation during redox cycling [20]. Conversely, more complex catalytic systems exploit the structural plasticity of Fe–S clusters to drive difficult chemical transformations that do not demand such rapid transfer of electrons. Notable examples include the P- and M-clusters of nitrogenase which undergo substantial, redox dependent, structural rearrangements during nitrogen fixation [21], and the proximal cluster of oxygen-tolerant [NiFe]-hydrogenases where a conformational change allows rapid recovery from O_2_ exposure [22, 23].

Alongside functional assays, a detailed understanding of the biochemistry of Fe-S clusters requires both structural and spectroscopic characterisation of the metalloprotein; UV-visible and electron paramagnetic resonance (EPR) spectroscopy are widely used. Typically, these analyses are performed on protein samples within very different physical and chemical environments, for example, spectroscopy is typically collected from protein solutions whereas structure determination requires the protein to be crystallised. In addition to placing the protein in the solid-state, the chemical conditions required for crystallisation are rarely similar to those experienced by the protein in solution. Further variation is introduced when temperature is considered: EPR measurements on iron sulfur proteins are usually performed at liquid helium temperatures, X-ray diffraction data are generally collected at liquid nitrogen temperatures and UV-visible spectroscopy data are typically collected under ambient conditions. Making any sort of correlation between redox related structural and spectroscopic changes relies on the assumption that the protein exhibits identical redox behaviour under these vastly different conditions.

In studies on hydrogenases, Vincent, Carr and coworkers have addressed this shortcoming by applying electrochemical poising to crystals of the enzyme, combined with use of infrared (IR) microspectroscopy to monitor the redox speciation of the catalytic site in crystallo, via the IR-active CO and CN^−^ ligands of hydrogenase [24–26]. Using electrochemically poised crystals for X-ray diffraction [26, 27] ensures both structure and spectroscopy are performed under the same physical and chemical conditions removing the sample-to-sample variation inherent to more conventional analyses. Since Fe-S proteins lack the intense, distinct vibrational bands necessary for IR analysis, just as they often lack strong electronic transitions required for resonance Raman spectroscopy, we turn to alternative spectroscopies (EPR and UV-visible) to monitor redox change in Fe-S protein crystals.

**Fig. 1:**
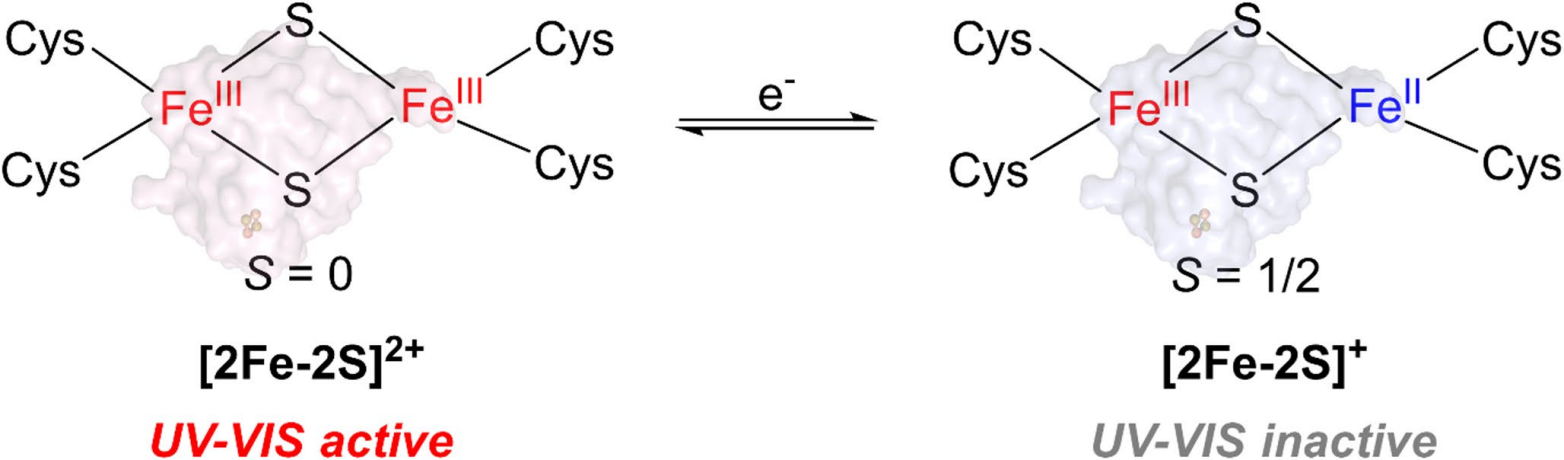
Redox scheme of spinach ferredoxin in its oxidised (left) and reduced (right) states. Details of charge distribution and electronic spin states of the cluster are also shown

**Fig. 2:**
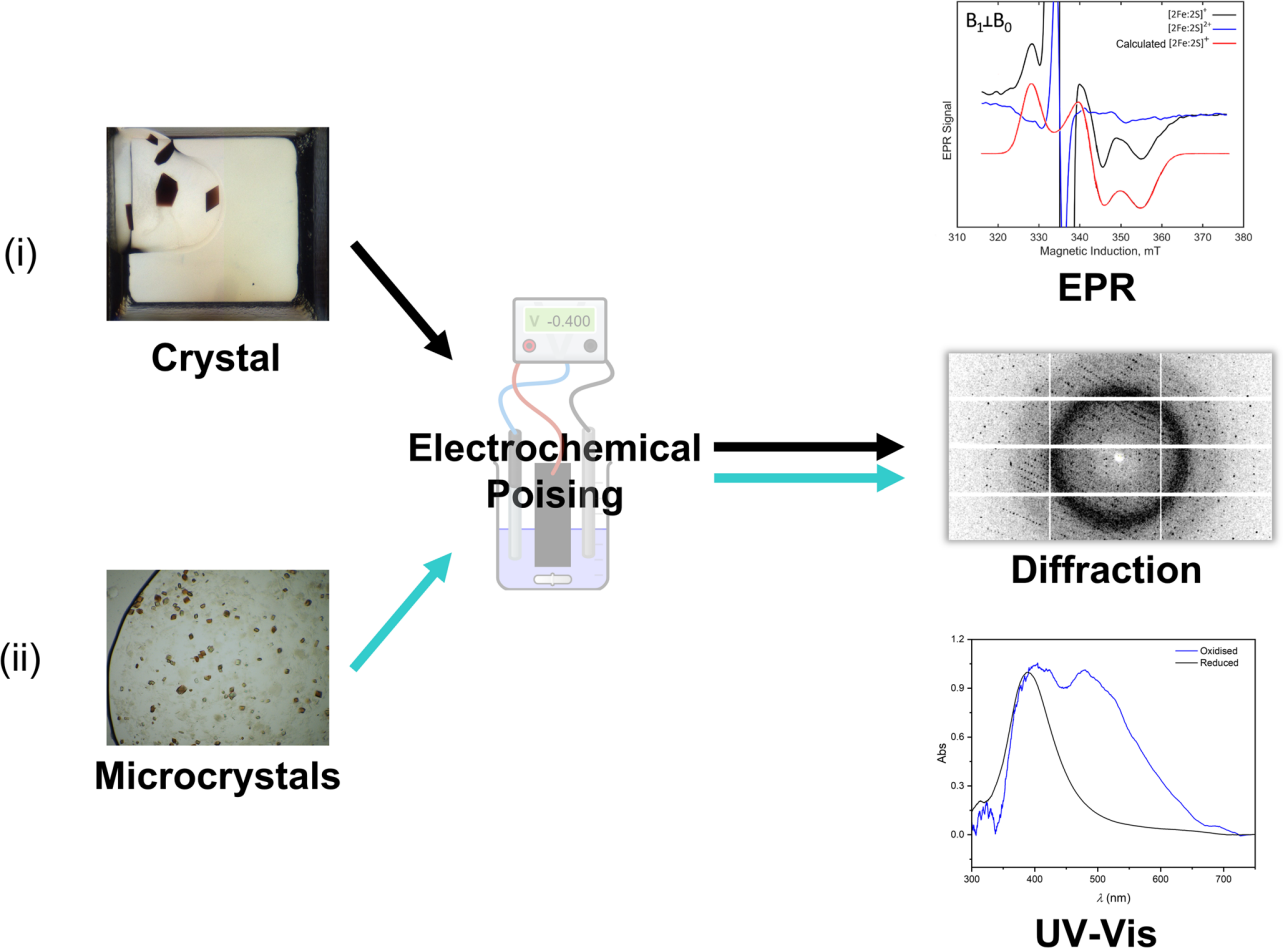
Overview of the electrochemical poising-microspectroscopy-structure platform described in this work. The process can be sub-divided into two distinct workflows (i) Larger crystals are electrochemically poised and then used to collect X-ray diffraction data and/or UV-visible spectra (black arrows). (ii) Microcrystal slurries are electrochemically poised at the same set of potentials before collection of EPR spectra (green arrows)

Here Ferredoxin I (Fdx) from *Spinacia oleracea* (spinach) is used as a model system to extend and validate the electrochemical poising platform, with in crystallo UV-visible and EPR spectroscopies employed to verify redox change, Fig. 1. Coupling electrochemical poising of crystals with established spectroscopies that are widely used for the characterisation of redox proteins, makes the poising pipeline more generalisable and will expand its appeal to a wide range of researchers in the field of bioinorganic chemistry. The model protein Fdx is a small, ~ 11 kDa, protein that contains a [2Fe-2S] cluster exhibiting distinct EPR and UV-visible spectroscopic signatures upon oxidation and reduction [28–30], that are suitable for validating the technique. This work aims to offer a complementary route for rigorous correlation of structural biology with spectroscopy, Fig. 2.

## Methods & materials

All chemicals used were purchased from Merck Life Sciences except EDTA-free protease inhibitor tablets that were obtained from Roche Molecular Biochemicals. The HisTrap Chelating HP column and size exclusion column were from Cytiva. All molecular biology reagents were purchased from New England Biolabs.

### Molecular biology

Previously constructed plasmid pGEX_Fd [31] expresses spinach ferredoxin I with an N-terminal GST-tag (Glutathione S-Transferase from *Schistoma japonicum*). The GST-FdI gene fusion was PCR amplified using primers GST_Fdx_for (GAATCCCCATGGCCATGTCCCCTATACTAGGTTATTGG) and GST_Fdx_rev (CATGCACTCGAGTTATGCGGTCAGTTCCTCTTCTTTGTGG) that introduced NcoI and XhoI sites at the 5’ and 3’ end of the PCR product, respectively. The PCR product and plasmid pETm11 were digested with NcoI and XhoI as per the manufacturer’s instructions with Plasmid pETm11 being further treated with shrimp alkaline phosphatase (following manufacturer’s protocol). The digested PCR product was then ligated into pETm11 placing a 6x histidine-tag and TEV protease cleavage site at the 5’ end of the GST gene to facilitate protein purification. The plasmid (Electronic Supplementary Information (ESI) Fig S1) was used to transform *E. coli* strain BL21 DE3 for protein expression.

### Protein expression and purification

Starter cultures were grown aerobically from single colonies, in LB medium supplemented with kanamycin (30 µg/mL) at 37 °C. These were then used to inoculate larger (1 L) cultures of LB/kanamycin to an OD_600_ of ~ 0.05. Bacteria were incubated aerobically at 37 °C in LB medium supplemented with kanamycin (30 µg/mL) until an OD_600_ of 0.3 was achieved, then the temperature was dropped to 18 °C. When an OD_600_ of 0.6 was reached, protein expression was induced by addition of IPTG (1 mM final concentration) and continuing incubation at 18 °C overnight.

Bacteria were harvested via centrifugation at 5000*g* for 10 min at 4 °C. The pelleted cells from 1 L culture were re-suspended in 20 mL of Buffer 1 (50 mM Tris pH 8 and 350 mM NaCl) supplemented by 10 g/mL DNase I 50 g/mL lysozyme and 1 tablet/L of complete EDTA-free protease inhibitor mixture. Cell lysis was performed using a cell disruptor (Constant systems, UK) with two passages at 28 kPSI at 4 °C. The resulting cell lysate was centrifuged at 40000*g* for 1 h at 4 °C. The resulting supernatant was loaded onto 5 mL HisTrap FF crude column pre-equilibrated in Buffer 1. After loading the column was washed with 10 column volumes of Buffer 1 to remove non-specifically bound proteins. The His-tagged ferredoxin was then batch eluted using Buffer 2 (50 mM Tris pH 8, 350 mM NaCl, 500 mM imidazole). The eluted ferredoxin was concentrated to a volume of 10 mL using a 30 kDa cut off spin concentrator (Amicon, UK).

The His-GST-tag was cleaved from Fdx by addition of 3 C protease to the protein solution, a step combined with overnight dialysis against 1 L of PreScission Buffer (150 mM NaCl, 50 mM Tris pH 8, 1 mM EDTA, 1 mM DL-dithiothreitol). The cleaved protein solution was reloaded onto the HisTrap Chelating HP column equilibrated with Buffer 1 and the cleaved ferredoxin protein fractions eluted were collected. The column was washed with an additional 1–2 column volumes of Buffer 1 to ensure elution of all cleaved ferredoxin. The eluted ferredoxin fractions were combined and concentrated to a volume of ~ 2 mL using a 5 kDa cut off spin concentrator (Amicon, UK) before loading onto an Superdex S75 size exclusion column (Cytiva UK) that had been pre-equilibrated with Buffer 3 (50 mM Tris pH 8 and 150 mM NaCl). All fractions containing purified ferredoxin, as judged by absorbance at 420 and 280 nm, were analysed by SDS PAGE before pooling, concentrating and re-applying to the Superdex S75 column. The second size-exclusion column was required to remove residual GST (presumably lacking the his-tag) that carried over from the re-application to the His-Trap column and the first round of size-exclusion chromatography. Fdx fractions were then pooled and concentrated as previously described to a final concentration of 30 mg/mL (calculated using E_420_ = 9400 cm^−1^M^− 1^). Purified Fdx was also analysed by protein mass spectrometry (Fig S2).

### Crystallisation and structural determination

Single crystals of Fdx for X-ray diffraction were grown aerobically using the sitting drop vapour diffusion method, using 96 well crystal plates (CrystalQuick X, Greiner, UK), and mixing 100 nL of Fdx (30 mg/mL) with an equal volume of crystallisation buffer (3.5 M ammonium sulfate, 0.10 M sodium phosphate pH 7.6–7.9). Drops were dispensed using a Mosquito crystallisation robot (SPT LabTech, UK) and left to equilibrate at 4 °C. Blood-red, diamond-shaped crystals appeared after 1 day, achieving maximum dimensions after a further 2–3 days (Fig S3). Crystals were transferred into 3.5 M ammonium sulfate, 0.10 M sodium phosphate pH 7.8, 5% (v/v) glycerol (hereon: ‘cryo-buffer’) and flash-cooled in liquid N_2_ prior to collection of X-ray diffraction measurements. Diffraction data were collected at beamline I24 (Diamond Light Source, UK) using an X-ray energy of 17.5 keV, 10 ms exposure and a Pilatus3 6 M detector (Table S1). Data reduction was performed using DIALS [32] and AIMLESS [33] within the Xia2 [34] pipeline. Initial phase estimates were generated by molecular replacement in Phaser [35] using PDB (9GYD) as a search model. The resulting maps were inspected in COOT [36] followed by iterative rounds of refinement in REFMAC5 [37] and manual rebuilding (COOT) until no further improvements were possible. This was followed by a final round of refinement in Phenix [38], with the quality of the resulting models assessed using Molprobity [39]. The resulting coordinates and structure factors have been submitted to the PDB with accession codes 9TXE (Oxidised), 29KI (Reduced −400 mV), 9TXQ (Reduced, -450 mV) and 9TXO (Re-Oxidised). Structural images were produced using PyMOL [40].

Microcrystals for EPR measurements and UV-visible microspectroscopy were grown similarly with slight changes in buffer ratios: 180 µL of crystallisation buffer was mixed with 50 µL of crystal seeds (also in crystallisation buffer) and 20 µL of protein at 30 mg/mL in a 1.5 mL Eppendorf tube before incubating at 4 °C. Microcrystals typically appeared after 48 h (Fig S4). The crystal seed stock was made by crushing single Fdx crystals using a glass “seed bead” kit (Hampton Research, USA) following the manufacturer’s protocol. The resulting 50 µL seed stock was diluted to a final volume of 150 µL before addition to the crystallisation experiments.

### Electrochemical poising

The methodology for electrochemical poising, spectroscopic verification and X-ray structure determination is illustrated schematically in Fig. 2.

All electrochemical manipulations were performed using a PalmSens4 potentiostat in cryo-buffer within a N_2_- filled glovebox (< 1 ppm O_2_), at 25 °C (ambient temperature of glovebox). After growing aerobically, all subsequent crystal manipulations were performed in the same anaerobic glovebox.

A three-electrode setup, consisting of a saturated calomel reference (PalmSens4), a carbon felt working electrode (Thermo Scientific, 99% pure, 7 cm × 31.8 mm × 3 cm), and a Pt wire counter electrode (Thermo Scientific, 99.95%, 12 cm × 0.5 mm) in a 0.5 M NaCl-filled fritted tube (6 mm OD), was used to poise methyl viologen (MV) at -400 and −450 mV (reduced) and 0 mV (re-oxidised). The MV solution was allowed to equilibrate with the electrode potential until the current stabilised close to zero. The open circuit potential of the poised buffer was then monitored for at least 1 h to ensure that a stable potential could be maintained (Figure S8). A midpoint potential of −400 mV was assumed for Fdx [28].

For X-ray diffraction and UV-visible spectroscopy, larger single crystals were transferred into 1 mL poised cryo-buffer (4 mM MV), incubated for 1 h at room temperature, and then flash-cooled in liquid nitrogen before measurement.

For EPR measurements, 1 mL of poised cryo-buffer containing 1 mM MV was added to an Eppendorf tube containing 100 µL of settled microcrystals and mixed by gentle aspiration until the suspension was uniform, followed by a soak of at least 1 h. Microcrystals were pelleted by gentle centrifugation (5 min, 9000*g*) then as much of the supernatant that could be removed without disturbing the microcrystal pellet was aspirated before the pellet was resuspended in 500 µL of freshly poised buffer. This buffer exchange process was repeated two further times before most of the supernatant was withdrawn, leaving ~ 100 µL buffer to resuspend the crystal pellet, 60 µL of which was loaded into a Q-band EPR tube using a gas-tight syringe (Hamilton, USA). The tube was sealed with vacuum grease, and the sample frozen in liquid nitrogen for subsequent EPR measurements.

### EPR spectroscopy

X-band EPR was acquired using an EMXmicro with a Premium bridge with Bruker BioSpin resonators ER4122SHQE-W1 for perpendicular mode. The sample temperature was controlled with an Oxford Instruments ITC-503 S and an ESR900 cryostat with cryogenic flow from a pressurised liquid helium dewar. Data were normalised according to temperature and spectrometer settings, with non-saturating conditions determined as follows: at 12 K, microwave power 80 µW, modulation amplitude 0.9 mT, with 4–8 sweeps of 300 s, averaged. Microwave frequencies were 9.3794(5), 9.3791(5), 9.3795(5), 9.3749(5), 9.3935(5) GHz for Fdx microcrystal slurry poised at -100, -200, -300, -400 and -450 mV. EPR spectra were subsequently processed, plotted in the MatLab (The Mathworks, Natick, NJ) environment and included EPR simulation using EasySpin [41] routines for MatLab. Presented spectra were obtained by subtracting signal from the corresponding buffer and mediator control samples.

### UV-visible spectroscopy

UV-visible absorption spectra were collected from cryo-cooled crystals at beamline I24 (Diamond Light Source, UK), using an on-line microspectrometer integrated into the end-station. The setup is comprised of two off-axis reflective objectives, a Shamrock 303i (Andor Technology) spectrometer, a Newton EM CCD detector and a fibre-coupled Xenon light source (Thorlabs) with a continuous spectrum over the wavelength range of 250–800 nm (Fig S5). Fdx crystals, at 100 K, were transferred to the goniometer. X-ray diffraction data and optical spectra were acquired concurrently from the same crystal volume. The spectra were an accumulation of 50 exposures of 8.8 ms duration each. The size of the white light beam was approximately 50 μm in diameter matching the size of the X-ray beam. UV-visible spectra were recorded before and after exposure to X-ray radiation and the data processed and plotted using OriginPro (2024. OriginLab Corporation, Northampton, MA, USA).

## Results & discussion

Electrochemical manipulation of protein crystals requires a pool of redox mediator(s) poised at a defined potential to equilibrate with redox centres within the crystal. To determine the feasibility of mediator ingress into Fdx crystals, and hence the likely success of electrochemical poising, solvent filled channels in crystals grown from as-isolated Fdx (9GYD) [31] were investigated using the LifeSoaks server [42] (Fig S6). This revealed a solvent channel radius of at least 4.8 Å (large enough for mediator passage) and significant exposure of the iron-sulfur cluster to the solvent channel.

The [2Fe-2S]^2+^ cluster of oxidised Fdx is diamagnetic and therefore EPR silent but exhibits characteristic UV-visible absorption bands at 420 nm and 469 nm arising from ligand-to-metal charge transfer (LMCT) [29]. In contrast, the one-electron reduced cluster, [2Fe-2S]^+^, bleaches at these wavelengths but is paramagnetic, displaying a characteristic rhombic *S* = 1/2 signal in EPR [43]. The reported mid-point potential of the [2Fe-2S] cluster is −400 mV vs. SHE [28, 31] but attempts to confirm this in the crystallisation buffer were unsuccessful because the high-salt conditions (3.5 M ammonium sulfate) induced immediate precipitation of Fdx consistent with the well-known behaviour of protein solutions in the presence of high concentrations of ammonium sulphate [44, 45]. The high-salt concentration of the medium from which Fdx crystals were grown also limits the choice of mediators due to poor solubility of organic redox dyes in this mixture. Four mediators were selected, with midpoints determined in the high-salt ‘cryo buffer’ determined by cyclic voltammetry (Fig S7, all potentials quoted vs. standard hydrogen electrode (SHE)) as follows: indigo carmine, (0 V), phenosafranin (-223 mV), neutral red (-344 mV) and methyl viologen (-476 mV). We were unable to find an organic mediator with sufficient solubility in the cryo-buffer which was able to hold a stable potential more negative than −450 mV, hence this was the lower limit for our crystal poising.

**Fig. 3:**
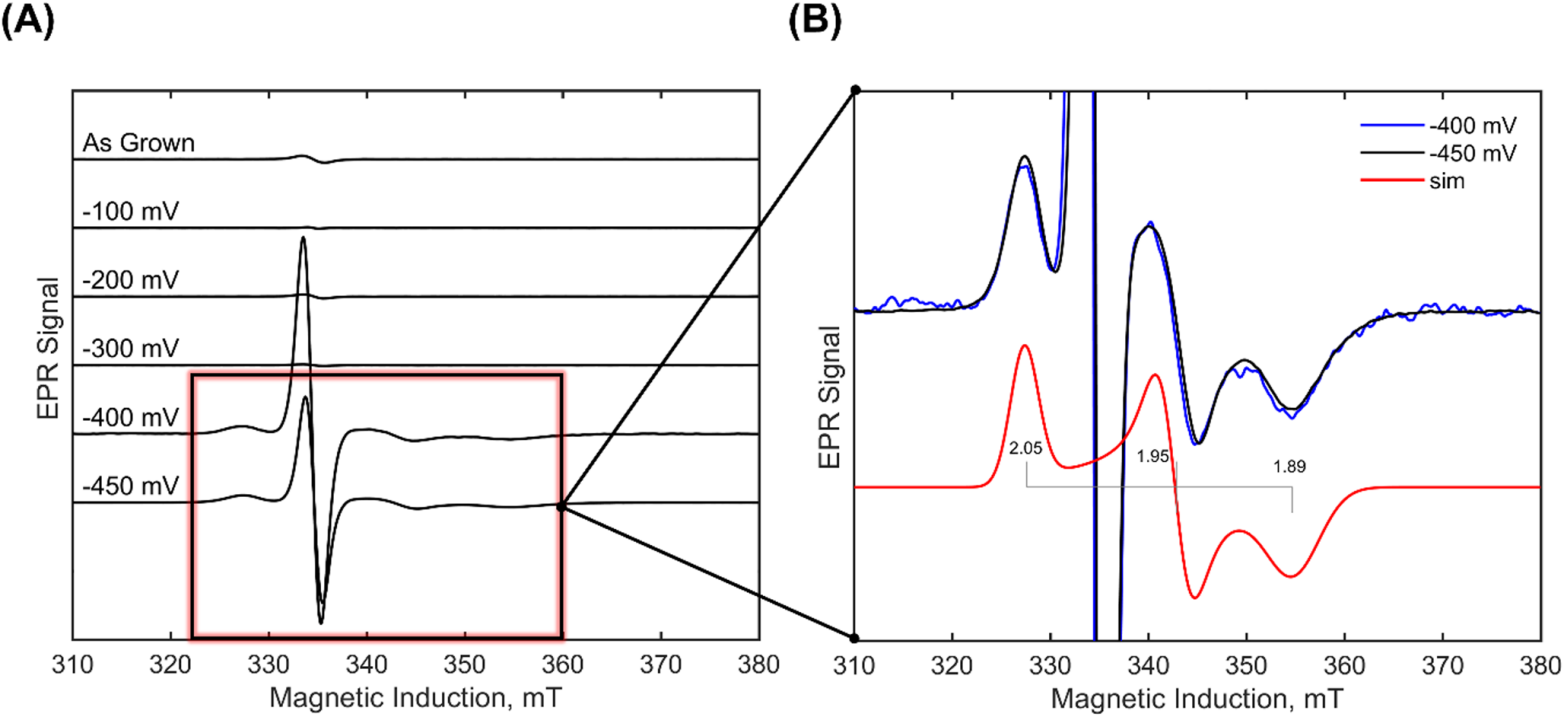
X band perpendicular-mode CW-EPR spectra of Fdx protein microcrystals **(A)** recorded at various applied potentials, under conditions described in Methods. **(B)** Inset: Fdx poised at -400 mV (blue) and −450 mV (black) and the simulation of the signal for reduced [2Fe-2S]^+^ species, g_1,2,3_ = 2.05, 1.95 and 1.89 (red)

An EPR redox titration was performed between 0 V and −450 mV on a slurry of Fdx microcrystals in the presence of the identified redox mediators to establish feasibility of manipulating Fdx redox state in crystallo (Fig. 3A). This employed a standard three-electrode setup to perform bulk electrolysis to poise mediator-enriched cryo-buffer at each desired potential. Assuming that the Fdx midpoint potential is unchanged in the high-salt cryo-buffer, by −450 mV Fdx should be 88% reduced according to the Nernst Equation. Additionally, the high ammonium sulfate concentration in the cryo-buffer shifts the mid-point potential of redox mediators to more positive potential (Fig S7), therefore a similar shift may also apply to Fdx meaning the protein is essentially completely reduced by -450 mV.

Microcrystal slurries were found to be more suitable for EPR spectroscopy than single crystals for two reasons; (i) the high protein concentration in crystals (~ 700 mg/mL; ~70 mM see SI) and the possibility to completely fill an EPR tube with microcrystals maximises signal to noise ratio; (ii) the random orientation of microcrystals within the tube masks any directional effects the crystals symmetry enforces on the paramagnetic species. This eliminates the need to measure spectra with the sample at multiple orientations, with respect to the applied magnetic field, which is necessary when measuring spectra from individual larger crystals [46]. On the other hand, quantitative EPR is more challenging when using microcrystal slurries since there is uncertainty in the density of the crystal suspension (crystals/volume) used for the EPR measurements.

The EPR spectrum of ‘As-Grown’ microcrystals shows no distinct EPR features, consistent with the cluster being in the oxidised [2Fe-2S]^2+^, EPR-silent state. Soaking microcrystals in buffer poised at -100 mV similarly showed no features characteristic of a reduced cluster. The potential was subsequently stepped more negative in 100 mV increments with no significant changes observed until the potential reached −400 mV. At this potential, in addition to the sharp radical signal from reduced methyl viologen (*g* = 2.0) [47, 48], a new set of features indicating a reduced [2Fe–2S]^+^ cluster was observed, with g-values (2.05, 1.95, 1.89) determined from spectral simulation agreeing with those previously reported (Fig. 3B) [30, 43]. A further 50 mV step to −450 mV, showed little change in intensity, indicative of full reduction of Fdx crystals by −450 mV in this buffer system. Poised buffer blanks were collected as controls (Fig S9) and used to subtract background contributions, however, difficulty in exact matching of concentrations meant that it was not possible to fully remove the signal arising from the reduced methyl viologen radical.

Fdx microcrystals poised at −450 mV were subsequently re-poised at 0 V. EPR analysis of this sample showed complete disappearance of the [2Fe–2S]^+^ *S* = 1/2 signal (Fig S10), consistent with full re-oxidation of the iron–sulfur cluster. This confirms feasibility of electrochemical manipulation of redox state in crystals of Fdx, with full reversibility, as we have previously observed for redox enzymes, FeFe and NiFe hydrogenases [27, 49]. The microcrystal slurries were also used for serial synchrotron X-ray diffraction measurements, generating a diffraction data set to a resolution of 1.7 Å (Table S1). Since larger cryogenically cooled crystals diffracted to much higher resolution (Table S2) all further diffraction data was collected from larger single crystals poised under identical conditions to the microcrystal slurries used for EPR.

**Fig. 4:**
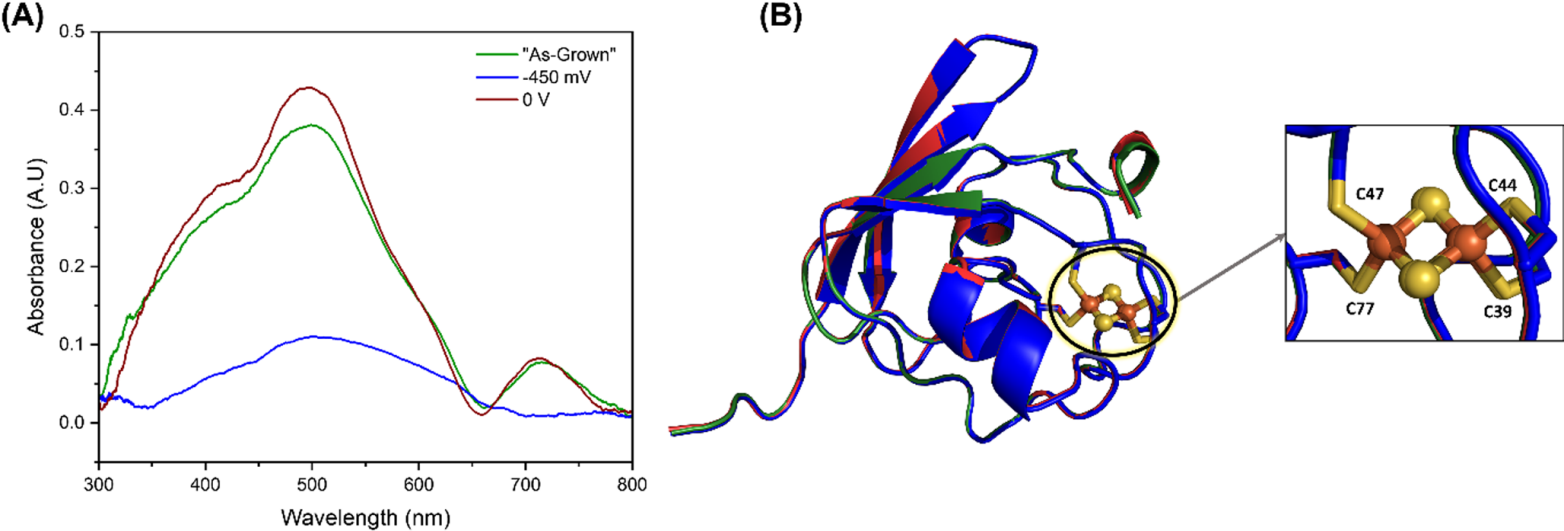
**(A)** In crystallo UV-visible spectra of Fdx crystals following X-ray exposure for oxidised ‘As-Grown’ Fdx (green), −450 mV poised Fdx (blue), and 0 V poised Fdx (maroon). **(B)** Superposed structures of “As-Grown” (green, 9TXE), -450 mV poised (blue, 9TXQ), and 0 V poised (maroon, 9TXO) of Fdx, with a close-up view of the [2Fe–2S] active site and coordinating cysteine residues bound to the iron centres

To correlate structure and spectroscopy for the two redox states of larger Fdx single crystals, X-ray diffraction (Table S2) with in situ UV-visible spectroscopy was conducted. Spectra collected before and after exposure to the X-rays were compared to ensure no photoreduction of the [2Fe-2S] cluster had occurred during the diffraction experiment. The UV-visible spectra collected for each crystal after exposure to X-rays are shown in Fig. 4A and the corresponding structures for “As-Grown” Fdx (green), -450 mV (blue) and 0 V (maroon) are shown in Fig. 4B. Oxidised spinach Fdx solution shows absorption peaks at 421 and 463 nm [50]. The “As-Grown” crystal shows absorbance in this region, as expected, although the peaks appear broader than in solution due a combinatorial effect of the variation in photons emitted by the xenon lamp used across the measured wavelength range (Fig S5) and the inherent challenges of in crystallo spectroscopy [51, 52], for example, scattering from the crystal can have a significant impact on measured signal intensity. Additionally, between 300 and 400 nm fewer photons are emitted from the Xe lamp, which combined with high sample absorption at these wavelengths diminishes signal for that region, giving rise to broader or flattened peaks or shoulders (Fig. 4A), rather than the sharper peaks observed for Fdx solution measured using the same microspectrometer (Fig S12). The peak at 470 nm, on the other hand, is enhanced due to the increased number of photons emitted from the Xe lamp between 450 and 550 nm.

Spectral bleaching in the −450 mV crystal confirms reduction of Fdx [29, 31] Some residual absorbance may be expected in the reduced sample due to the methyl viologen radical (Fig S13), although concentration of the mediator was only 4 mM, compared to an effective concentration of Fdx in crystals of ca. 70 mM. Recovery of signal in the 0 V crystal confirms re-oxidation. The high effective concentration of Fdx in crystals presumably accounts for observation of an additional low-energy absorbance peak around 720 nm in both oxidised crystals, absent in the reduced sample. These data are in good agreement with spectra performed on solutions of reduced and oxidised Fdx performed on the same instrument. Oxidised methyl viologen has no significant absorption above 400 nm and hence should not contribute to the spectra of the oxidised samples (Fig S13). Although it is not possible to confirm complete reduction of Fdx in the −450 mV poised crystals, together, these results suggest a high level of reduction of Fdx in this sample.

In addition to the capability to simultaneously measure UV-visible and X-ray diffraction from the same crystal at a synchrotron, it is possible to perform dynamic spectral measurements for the duration of diffraction data collection. This enables real-time confirmation that datasets collected on the differently treated Fdx crystals were not suffering from photoreduction upon exposure to X-ray radiation. It was found that collection of diffraction data in small (20°) wedges, rather than a single continuous rotation, was necessary to prevent photoreduction of the metal cluster.

A superposition of X-ray structures obtained from the same crystals is shown in Fig. 4B, and shows minimal structural change between redox states, consistent with structures from chemically-reduced and re-oxidised Fdx [31]. Comparison of all atoms in the structure for the “As Grown” (oxidised) crystal with the those in the −450 mV (reduced) and 0 mV (re-oxidised) structures gave Root-Mean-Square Deviations of 0.065 Å and 0.072 Å, respectively. Such structural rigidity would aid efficient electron transport by minimising reorganisation energy upon redox change. A detailed comparison of the bond lengths of coordinating cysteines and sulfur atoms to both iron centres (Table S3) shows minimal differences that are within the coordinate error of the structural models. This is also consistent with observations from ultra-high-resolution structures reported for HiPIP iron-sulphur protein [17, 18]. 

## Conclusion

We have shown how single crystals and microcrystals of Fdx can be driven reversibly between oxidised and reduced states by application of an external electrochemical potential, resulting in negligible loss in diffraction quality of the crystals. Importantly, this shows that our approach to electrochemical poising of protein crystals can be extended beyond hydrogenases. EPR and UV–visible spectroscopy were used to verify the redox state of the [2Fe–2S] cluster in Fdx. Samples were electrochemically poised at room temperature under identical conditions and subsequently flash-cooled in liquid nitrogen for measurement. The use of Fdx crystals harmonised sample state across techniques, allowing online UV–visible spectroscopy and X-ray diffraction data to be collected simultaneously, under identical cryogenic conditions (100 K). EPR spectra were acquired under cryogenic liquid helium conditions (10 K), to obtain resolved line shapes from [Fe-S] clusters which are prone to broadening at liquid nitrogen temperatures [53]. Protein structures determined from X-ray diffraction data collected below 20 K have proved that lower temperatures have minimal impact on overall protein structure compared to data collected at 100 K, and do not affect subsequent biochemical interpretation [54, 55]. Online UV–visible monitoring during X-ray data collection demonstrated that the redox state was controlled exclusively by electrochemical poising and was not compromised by X-ray-induced photo-reduction. Overall, this study demonstrates a robust pipeline for precise poising of redox state in protein crystals, spectroscopic verification, and high-quality X-ray data collection, which should have wide applicability in mechanistic and structure function studies of electron transport proteins and redox enzymes.

## Supplementary Information

Below is the link to the electronic supplementary material.

Supplementary Material 1

## Data Availability

Coordinates and structure factors describing Oxidised (“As-Grown”), Reduced (-400 mV), Reduced (-450 mV) and Re-Oxidised (0 V) Fdx have been deposited in the Protein Data Bank with accession codes 9TXE, 29KI, 9TXQ and 9TXO respectively. Further data supporting the findings of this study are available within the paper and its Electronic Supplementary Information files. Should any raw data files be needed in another format they are available from the corresponding authors upon reasonable request.
