# Supplementary material for: Crystallise, poise, capture: a multimodal platform for correlated structural and spectroscopic characterisation of redox enzymes
Source: J Biol Inorg Chem. 2026 May 5;31(3):153–62. doi: 10.1007/s00775-026-02148-x (PMC13287116; doi:10.1007/s00775-026-02148-x)
Supplement: Supplementary file 1 — Supplementary Material 1 [file 775_2026_2148_MOESM1_ESM.pdf]

Crystallise, Poise, Capture: A Multimodal Platform for Correlated Structural and Spectroscopic Characterisation of Redox Enzymes

Shoba Laxmi [1, 2], Sofia Jaho [3], William K. Myers [4], Kylie A. Vincent [1, *], Stephen B. Carr [1, 2, *]

[1]Department of Chemistry, University of Oxford, Inorganic Chemistry Laboratory, South Parks Road, Oxford, UK

[2]Research Complex at Harwell, Rutherford Appleton Laboratory, Harwell Campus, Didcot, UK

[3]Diamond Light Source, Harwell Science and Innovation Campus, Didcot, UK

[4]Centre for Advanced Electron Spin Resonance (CAESR), University of Oxford, Oxford, UK

Supporting Information

Table Of Contents

Plasmid Design 2

Effective Crystal Protein Concentration Calculation 2

Mass Spectrometry of Purified Fdx 3

Size Comparison of Single Crystals and Microcrystals 4

UV-Visible Spectrum of Xenon Lamp 5

Solvent Channel Analysis 6

Electrochemistry – Cyclic Voltammetry of Mediators 7

Electrochemistry – Open Circuit Potential (OCP) Monitoring of Poised Methyl Viologen 7

Electron Paramagnetic Resonance (EPR) 8

X-Ray Diffraction 10

Superimposition of -400 mV and -450 mV Poised Fdx Structure 12

UV-Visible Spectroscopy of Fdx Solution (Microspectroscopy) 14

UV-Visible Spectroscopy of Poised Mediators 15

References 16

Plasmid Design

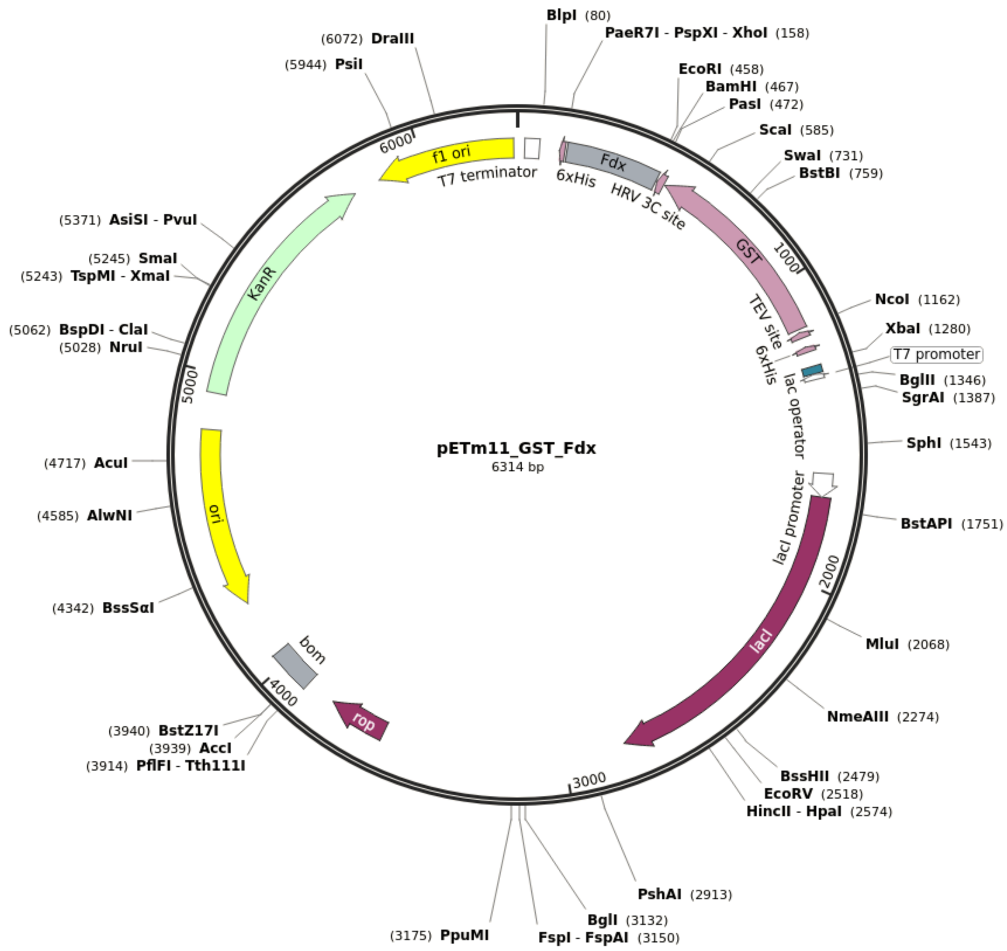

Figure S1. Plasmid map design of modified Fdx expression plasmid. Fdx is expressed as a GST fusion, to aid solubility, with N-terminal 6 x His-tag attached for ease of purification.

Effective Crystal Protein Concentration Calculation

In a Fdx crystal of $P2_12_12_1$ space group, there are 4 asymmetric unit (ASU) per unit cell and each ASU contains 1 molecule of Fdx. Therefore, a crystal contains:

Number of moles in a crystal unit cell: $\frac{4}{6.0221408 \; x \; 10^{23}} = 6.64 \; x \; 10^{-24}$ moles

Volume of the unit cell: $(30.265 \text{ Å} \; x \; 51.21 \text{ Å} \; x \; 61.17 \text{ Å}) \; x \; 10^{-27}$

$= 9.48 \; x \; 10^{-23}$ L

Therefore, concentration in a Fdx crystal $= \frac{6.64 \; x \; 10^{-24}}{9.48 \; x \; 10^{-23}} = 0.07$ M

$= 70$ mM

Mass Spectrometry of Purified Fdx

(i)

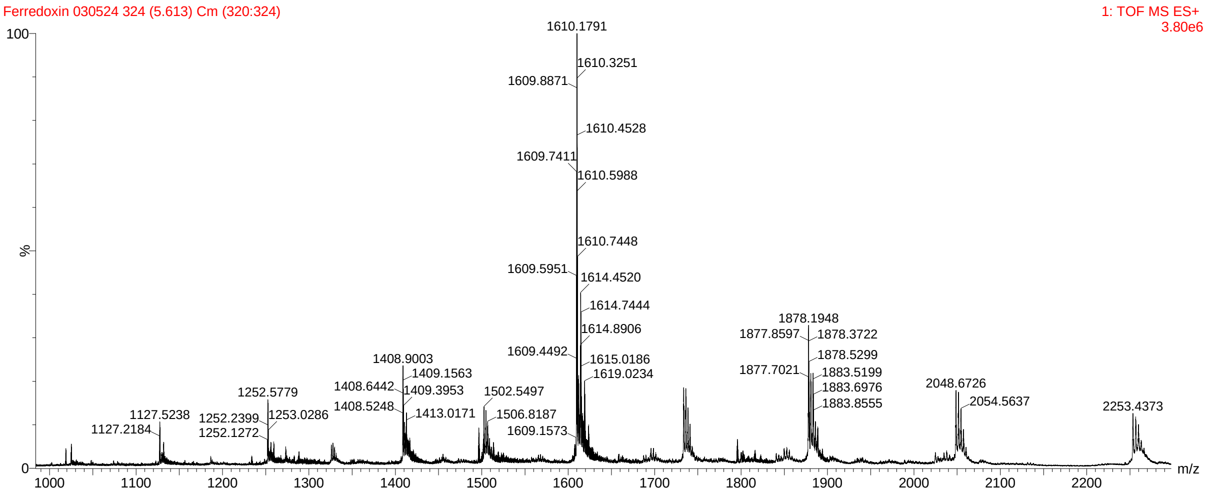

(ii)

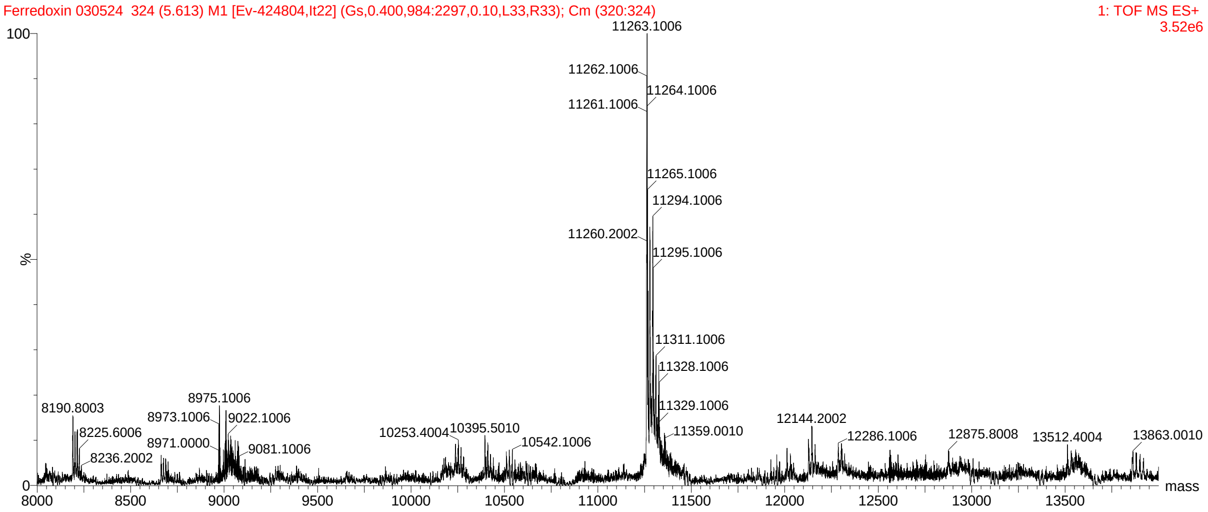

Figure S2. Mass Spectrometry of purified Fdx after cleavage of His-GST fusion tag. (i) Full m/z spectrum (ii) Deconvoluted mass spectrum showing a peak with molecular weight of 11263 Da corresponding to Fdx.

Size Comparison of Single Crystals and Microcrystals

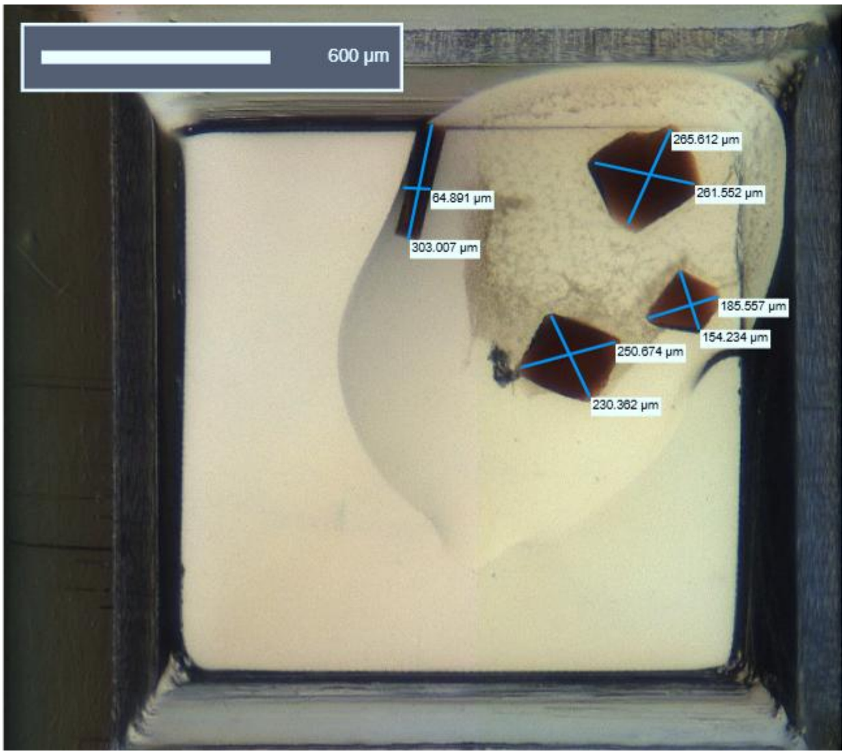

Figure S3. Image of Fdx crystals used for X-ray diffraction and UV-Visible spectroscopy with dimensions indicated. Crystals were visualised using Formulatrix® crystal imager.

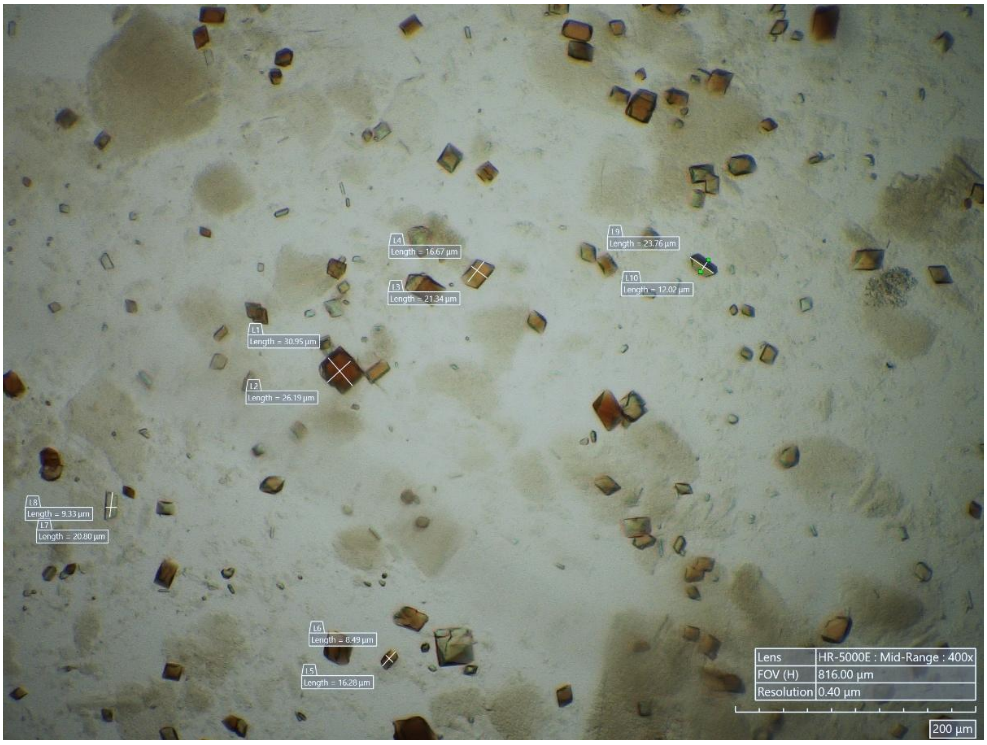

Figure S4. Image of microcrystal slurry of Fdx, visualised using Hirox Microscope at 400x magnification. Crystal dimensions are indicated above in white and range from 8.50 to 30.9 µm.

UV-visible Spectrum of Xenon Lamp

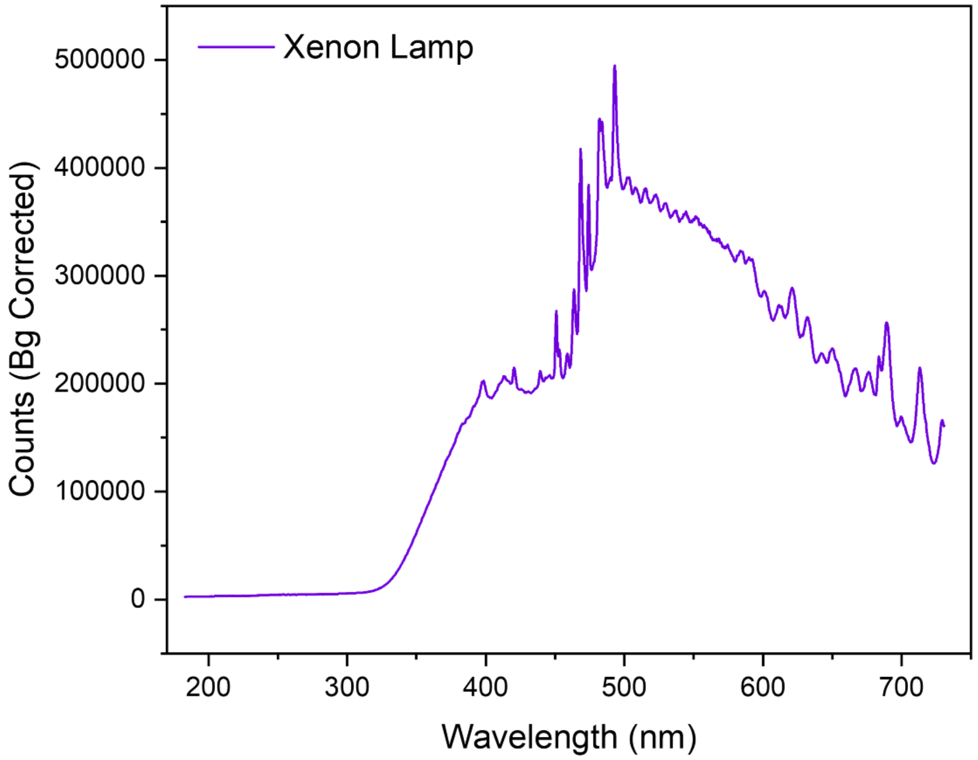

Figure S5. UV-visible reference spectrum of the Xenon lamp used with the on-line microspectrometer at beamline I24 (10 ms exposure, 10 accumulations).

Solvent Channel Analysis

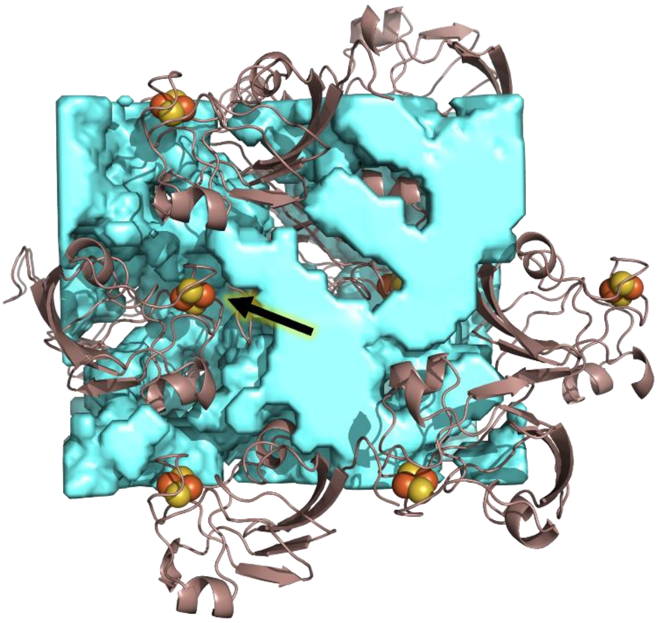

Figure S6. Solvent channels (cyan) in the crystal lattice visualised using the LifeSoaks server[1] Fdx is coloured pale red and the redox active [2Fe-2S] cluster shown as spheres. The narrowest channel radius is calculated to be 4.80 Å and the black arrow highlights the proximity of the cluster to the solvent channel. This facilitates electron transfer from poised mediators that diffuse into the crystal via these channels.

Electrochemistry – Cyclic Voltammetry of Mediators

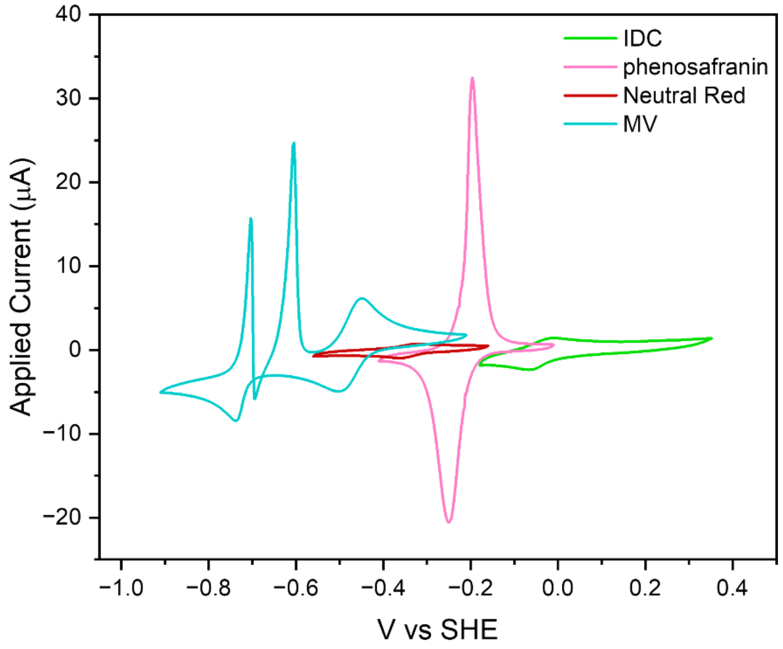

Figure S7. Cyclic voltammetry of selected mediators (each at 1 mM) in Fdx cryo-buffer, recorded at 20 mV/s scan rate at room temperature using a glassy carbon working electrode, where IDC = indigo carmine and MV = methyl viologen.

Electrochemistry – Open Circuit Potential (OCP) Monitoring of Poised Methyl Viologen

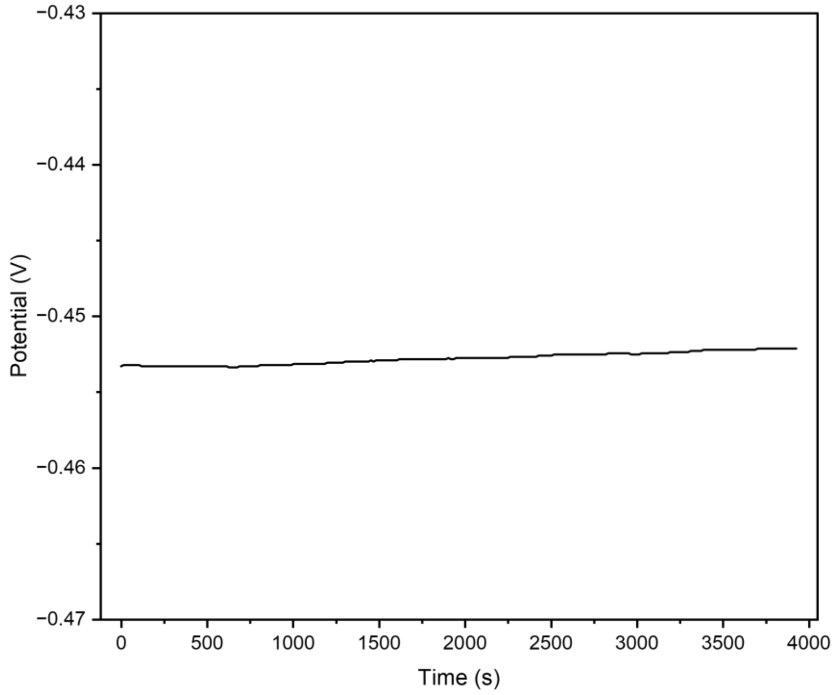

Figure S8. OCP of poised methyl viologen at -450 mV (vs SHE) over the duration of approximately an hour.

Electron Paramagnetic Resonance (EPR)

Accurate determination of protein concentration in microcrystal slurries is not straightforward, unlike in solution, hence spin quantification was not performed in this work.

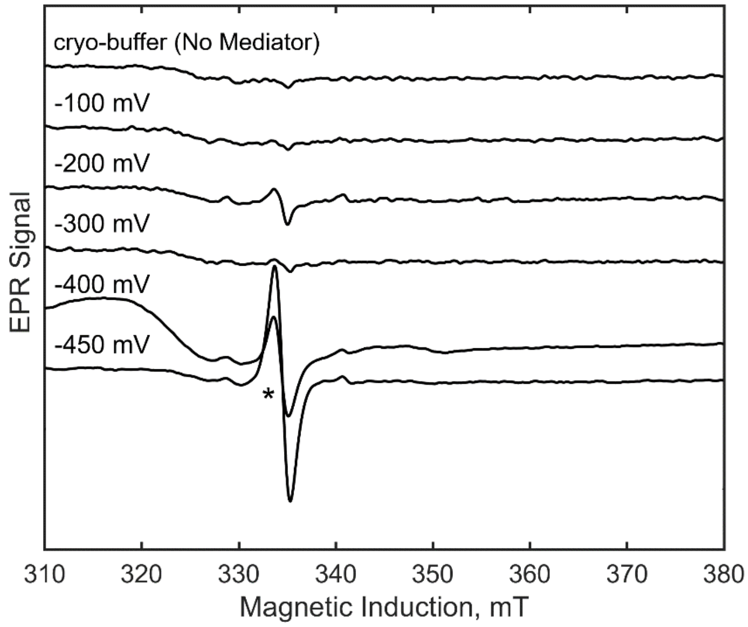

Figure S9. Control X-band perpendicular mode CW-EPR spectra used for subtraction of buffer and mediator signals (outlined in Fig 3 in the main text). The minor feature present at -200 mV and -300 mV are radical signals arising from trace amounts of carbon felt fibres (that have shed from the working electrode) present in buffer solution. The intense signal at $g = 2$ (*) in the -400 mV and -450 mV spectrum is due to reduced methyl viologen.[2, 3] The deviation in baseline for -400 mV buffer spectrum is due to difference in resonator background and not arising from the buffer.

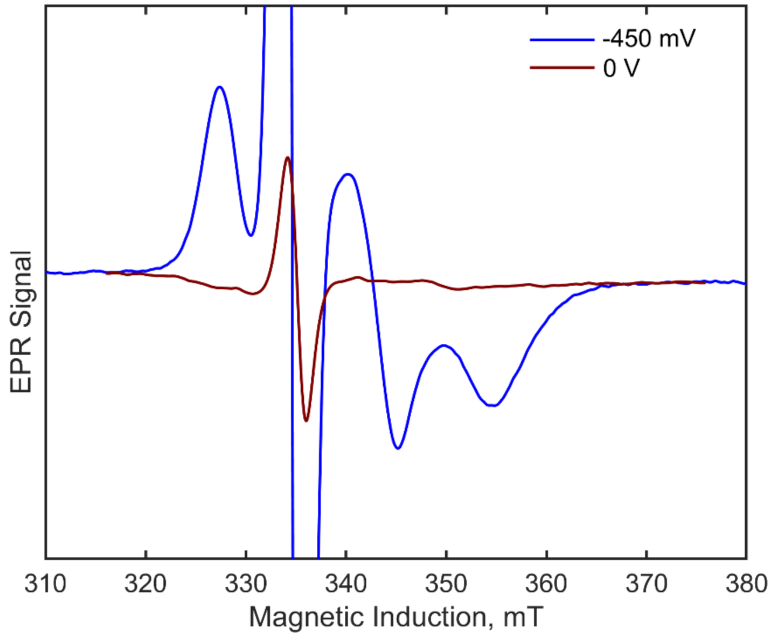

Figure S10. X-band perpendicular mode CW-EPR of Fdx microcrystal slurry electrochemically reduced at -450 mV and 0 V (vs SHE) in blue and maroon, respectively, under conditions detailed in Methods section, 2.5 of the main text. The signal approximately $g = 2.0$ is attributed to adventitious contributions from carbon felt fibres[4] used in the electrochemical setup.

X-Ray Diffraction

Table S1. X-ray data collection statistics for “As-Grown” Fdx microcrystals collected at I24 serial crystallography beamtime.

Species	As-Grown
Space group	$P2_1 2_1 2_1$
Unit cell dimensions (Å)	a= 30.353<br>b= 62.16<br>c= 52.516
Resolution (Å)	62.16 – 1.70<br>(1.73 – 1.70)
Total reflections	261485<br>(7739)
Unique reflections	11493 (567)
Completeness (%)	99.99 (100)
Multiplicity	22.8 (13.6)
<I/s>	4.8 (1.0)
R_{merge} (%)	0.725 (7.30)
R_{pim} (%)	0.119 (2.033)
$CC_{1/2}$	0.939 (0.205)

Values in parenthesis refer to highest resolution shell

Table S2. X-ray data collection and refinement statistics for Fdx poised at each potential.

**PDB code**	**9TXE**	**29KI**	**9TXQ**	**9TXO**
Potential vs SHE	-	-400 mV	-450 mV	0 V
Species	Oxidised [2Fe-2S]$^{2+}$	Reduced [2Fe-2S]$^{+}$	Reduced [2Fe-2S]$^{+}$	Re-Oxidised [2Fe-2S]$^{2+}$
Space group	$P2_1 2_1 2_1$	$P2_1 2_1 2_1$	$P2_1 2_1 2_1$	$P2_1 2_1 2_1$
Unit cell dimensions (Å)	a= 30.265<br>b= 51.212<br>c= 61.169	a= 30.02<br>b= 51.55<br>c= 61.09	a=30.220<br>b=51.770<br>c=61.380	a=30.232<br>b=51.756<br>c=61.309
Resolution (Å)	30.58 - 0.95<br>(0.98 - 0.95)	30.54 – 1.11<br>(1.13 – 1.11)	39.57 - 1.10<br>(1.12-1.10)	39.54 – 1.20<br>(1.22 – 1.20)
Total reflections	371983 (15429)	470734 (13557)	522373 (25435)	436323 (20917)
Unique reflections	59899 (3860)	38111 (1693)	39926 (1964)	30858 (1484)
Completeness (%)	98.68 (83.95)	99.60 (93.40)	100.0 (100.0)	100.0 (100.0)
Multiplicity	6.1 (5.2)	12.4 (8.0)	13.1 (13.0)	14.1 (14.1)
<I/s>	11.3 (0.7)	12.4 (0.8)	11.7 (0.8)	11.8 (0.4)
R_{merge} (%)	0.056 (1.490)	0.077 (2.243)	0.080 (4.240)	0.090 (2. 694)
R_{pim} (%)	0.025 (0.719)	0.023 (0.803)	0.023 (1.223)	0.025 (0.742)
$CC_{1/2}$	0.999 (0.489)	0.999 (0.257)	0.999 (0.421)	0.999 (0.425)
**Refinement**				
R_{work}/R_{free} (%)	0.1642 / 0.1784<br>(0.3958 / 0.4007)	0.1656 / 0.1942<br>(0.3351 / 0.3354)	0.1670 / 0.1851<br>(0.3360 / 0.3455)	0.1831 / 0.2160<br>(0.3656 / 0.3544)
No. of atoms	871	858	860	833
Macromolecule	777	777	785	777
Solvent	90	77	71	52
**Average B-factors**				
Macromolecule	14.51	19.39	20.70	21.22
Solvent	22.84	26.50	27.37	27.60
RMSD bond lengths (Å)	0.006	0.007	0.009	0.008
RMSD angles (°)	0.90	0.93	1.08	0.98
Ramachandran plot favoured / outliers (%)	98.98/0	98.89/0	97.96/0	98.98/0
Rotamer Outliers (%)	0	0	1.10	0
Clashscore	0	0	1.31	0
Molprobity Score	0.5	0.5	0.9	0.5

Values in parenthesis refer to highest resolution shell

Superimposition of -400 mV and -450 mV poised Fdx Structure

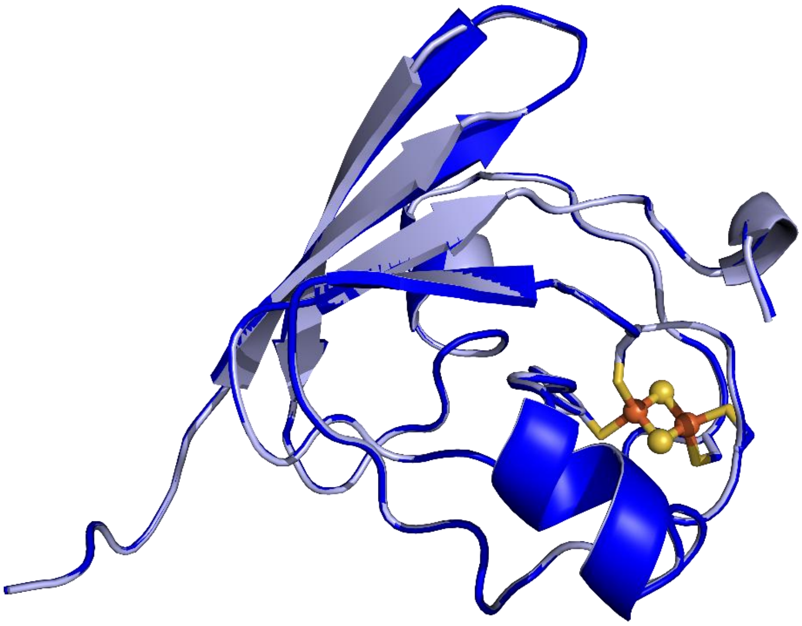

Figure S11. Root-Mean-Square-Deviation (RMSD) of -400mV poised Fdx structure is 0.06 with respect to -450 mV reduced structure. (Blue) -450 mV reduced structure and (Light Blue) -400 mV reduced structure, showing structural rigidity between the two potentials.

Table S3. Selected bond lengths and angles of the [2Fe-2S] cluster in the Oxidised (9TXO), Reduced -400 mV (29KI), Reduced -450 mV (9TXQ) and 9TXE (Re-Oxidised).

**Bond Length (Å)**	Oxidised (“As-Grown”)	Reduced (-400 mV)	Reduced (-450 mV)	Re-Oxidised (0 V)
S1 – Fe1	2.26	2.27	2.28	2.25
S2 – Fe1	2.22	2.20	2.24	2.22
C39 – Fe1	2.31	2.30	2.32	2.31
C44 – Fe1	2.29	2.27	2.28	2.29
S1 – Fe2	2.22	2.21	2.23	2.21
S2 – Fe2	2.18	2.15	2.20	2.18
C47 – Fe2	2.29	2.28	2.30	2.27
C77 – Fe2	2.30	2.31	2.31	2.28
**Bond Angle (°)**	Oxidised (“As-Grown”)	Reduced (-400 mV)	Reduced (-450 mV)	Re-Oxidised (0 V)
C44 – Fe1 – S2	120.74	121.36	121.17	120.76
C39 – Fe1 – S1	120.32	120.07	120.43	121.78
C44 – Fe1 – C39	104.23	104.12	103.62	103.31
S2 – Fe1 – S1	102.64	101.60	101.77	101.75
C47 – Fe2 – S2	111.24	111.30	110.62	111.74
C77 – Fe2 – S1	117.24	116.92	117.79	117.70
C47 – Fe2 – C77	108.11	107.67	107.85	107.92
S2 – Fe2 – S1	105.09	105.26	104.75	104.16

UV-visible Spectroscopy of Fdx Solution (Microspectroscopy)

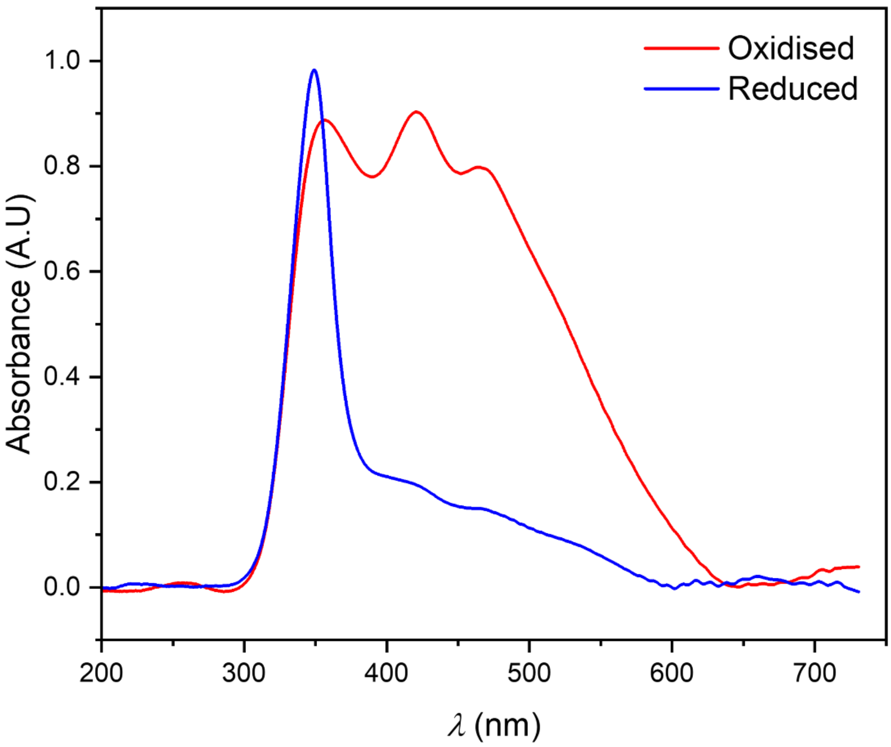

Figure S12. Solution spectra of 1 µL drop of 2 mM "As-Grown" and dithionite reduced Fdx measured on the off-line I24 microspectrophotometer using exposure of 10 ms and 10 accumulations per spectrum.

UV-visible Spectroscopy of Poised Mediators

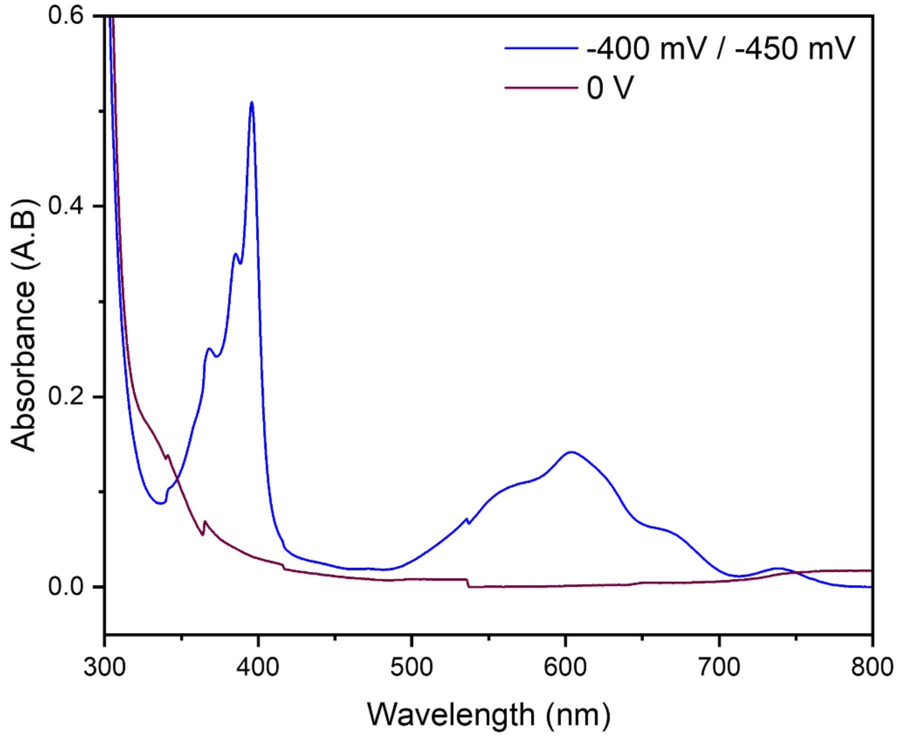

Figure S13. UV-visible spectrum of 1 mM of electrochemically poised methyl viologen in the cryo-buffer at -400 mV/-450 mV (blue) and 0 V (maroon), measured at room temperature using a Shimadzu 1800 spectrophotometer.

References

[1] J. Pletzer-Zelgert, C. Ehrt, I. Fender, A. Griewel, F. Flachsenberg, G. Klebe and M. Rarey (2023) Acta Crystallographica Section D 79:837–856

[2] J. A. Herder, S. J. Kruse, A. D. Nicholas, T. Z. Forbes, E. D. Walter, H. Cho and C. L. Cahill (2024) Inorganic Chemistry 63:4957–4971

[3] R. Zhan, S. Song, Y. Liu and S. Dong (1990) Journal of the Chemical Society, Faraday Transactions 86:3125–3127

[4] A. L. Kovarskii, V. V. Kasparov, A. V. Krivandin, O. V. Shatalova, R. A. Korokhin and A. M. Kuperman (2017) Russian Journal of Physical Chemistry B 11:233–241
